# Sympathetic Biomarker Dynamics Post-Myocardial Infarction: TH, PGP9.5, and SYN Expression Discordance in Murine Hearts

**DOI:** 10.3390/ijms26199456

**Published:** 2025-09-27

**Authors:** Tianshui Yu, Baoqing Pei, Dong Zhao

**Affiliations:** 1 Key Laboratory of Evidence Science, China University of Political Science and Law, Ministry of Education, Beijing 100088, China; zhaod@cupl.edu.cn; 2 Beijing Key Laboratory for Design and Evaluation Technology of Advanced Implantable & Interventional Medical Devices, Beijing Advanced Innovation Center for Biomedical Engineering, School of Biological Science and Medical Engineering, Beihang University, Beijing 100083, China; pbq@buaa.edu.cn

**Keywords:** myocardial infarction (MI), sympathetic remodeling, tyrosine hydroxylase (TH), protein gene product 9.5 (PGP9.5), synaptophysin (SYN)

## Abstract

Myocardial infarction (MI) and its sequelae continue to be the leading cause of mortality globally. Following MI, a series of structural pathophysiological changes occur in the myocardium, including sympathetic remodeling. Tyrosine hydroxylase (TH), protein gene product 9.5 (PGP9.5), and synaptophysin (SYN) are recognized as key markers of sympathetic nerves. However, the expression patterns of these biomarkers during sympathetic remodeling, particularly their temporal profiles, remain insufficiently characterized. A cohort of 60 healthy adult male C57BL/6 mice was randomly divided into a control group (*n* = 12) and four MI groups with postoperative intervals of 2, 5, 7, and 10 days (*n* = 12/group). MI was induced via permanent ligation of the left anterior descending coronary artery (LAD). Cardiac tissues were subjected to histological analyses (HE and Masson’s trichrome staining), immunohistochemical profiling, and quantitative reverse-transcriptase PCR (qRT-PCR) (TH, PGP9.5, and SYN). Immunohistochemical staining revealed that TH-, PGP9.5-, and SYN-immunopositive sympathetic nerves were present in the epicardium, myocardial interstitium, and the periphery of small blood vessels in normal mice. Normal cardiomyocytes were negative for TH but exhibited focal expression of PGP9.5 and SYN. In the myocardial infarction tissue, TH-positive staining indicated sympathetic nerve proliferation in the epicardium, myocardial infarction border zone, and infarct zone, with peak expression occurring at 7 days post-MI. In contrast to TH, PGP9.5 exhibited prominent immunoreactivity, specifically localized to the infarct core and peri-infarct zone cardiomyocytes, while SYN was primarily located in fibroblast-like cells within the same region. qRT-PCR analyses revealed that the time-dependent trends of TH, PGP9.5, and SYN mRNAs exhibited similarities, peaking between 5 and 7 days post-MI. TH demonstrates higher specificity than PGP9.5 and SYN in sympathetic nerve identification, solidifying its role as the optimal biomarker for post-MI sympathetic remodeling. The ectopic expression of PGP9.5 and SYN in non-neuronal cells within myocardial infarction tissue remains speculative and requires further mechanistic studies for validation.

## 1. Introduction

Cardiovascular diseases, particularly myocardial infarction (MI), remain the leading cause of global mortality. Post-MI myocardial tissue undergoes pathological sympathetic remodeling, characterized by a biphasic pattern: an initial denervation phase due to ischemic nerve terminal damage, followed by maladaptive hyperinnervation within the infarct border zone. This remodeling manifests as regional sympathetic hyperactivity mediated through nerve growth factor (NGF)-dependent sprouting and inflammatory cytokine-driven neurogenesis [1]. The denervation phase involves the rapid degradation of sympathetic terminals, evidenced by a >70% reduction in synaptic vesicle proteins, e.g., synaptophysin (SYN), and disrupted neurotransmitter release machinery. Subsequently, macrophage-derived NGF and matrix metalloproteinases (MMPs) trigger axonal sprouting, leading to dysregulated reinnervation with excessive tyrosine hydroxylase (TH)-positive fibers. Crucially, this hyperinnervation exhibits spatial heterogeneity: chondroitin sulfate proteoglycans (CSPGs) in the cardiac scar prevent sympathetic reinnervation by binding the neuronal protein tyrosine phosphatase receptor sigma (PTPσ), leading to permanent denervation in the infarct core [2]; while the border zone develops nerve density gradients that create electrophysiological dispersion [3]. Sympathetic remodeling is strongly implicated in the pathogenesis and progression of post-infarct malignant arrhythmias (MAs), which may precipitate lethal complications, including heart failure (HF) and sudden cardiac death [4].

TH, the rate-limiting enzyme in catecholamine biosynthesis, is predominantly localized to catecholaminergic neurons [5], sympathetic ganglia [6], and sympathetic nerve terminals [7]. The angiotensin II-angiotensin II receptor type 1 (AT1R) axis significantly upregulates tyrosine hydroxylase (TH) expression and enhances axonal trafficking of TH mRNA, leading to increased local norepinephrine synthesis within hyperinnervated areas, which critically contributes to electrophysiological instability and arrhythmogenesis [8]. TH also functions as a significant biomarker in diagnosing conditions such as pheochromocytoma [9] and Parkinson’s disease [10]. Protein gene product 9.5 (PGP9.5), also referred to as ubiquitin carboxy-terminal hydrolase L1 (UCHL1), is a specific protein found in nerve fibers. It is extensively distributed in neurons [11] and nerve fibers [12] within both the central and peripheral nervous systems. SYN is localized in the membrane of presynaptic vesicles within nerve terminals in both the central and peripheral nervous systems [13]. SYN plays a crucial role in the import, transport, and release of neurotransmitters from synaptic vesicles by facilitating Ca^2+^-dependent membrane fusion, which is closely associated with nerve growth, repair, and regeneration [14,15]. Previous studies have identified the expressions of TH, PGP9.5, and SYN in MI [16,17,18]. However, the time-dependent expressions of TH, PGP9.5, and SYN, as well as their differential distribution during MI, remain undefined. Consequently, it is essential to identify a more suitable indicator for determining sympathetic nerve presence in the myocardial tissue and to discuss the mechanism by which TH, PGP9.5, and SYN contribute to the progression of MI.

This investigation systematically characterizes the temporal dynamics and spatial heterogeneity of TH, PGP9.5, and SYN in the post-infarct myocardium, preliminarily exploring their distinct roles in the pathogenesis of MI.

## 2. Results

### 2.1. Microscopic Analysis of MI Tissue in Mice

**Figure 1:**
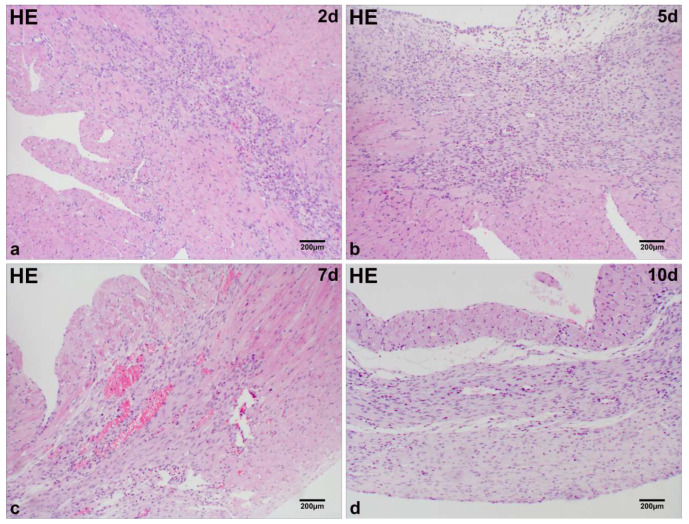
Histological characterization of MI in mice. Single-nucleated cell infiltration and fibroblast-like cell proliferation dominate the infarct region at day 2 post-MI (**a**). Fibroblast-like cells persist with sparse regenerating cardiomyocytes at days 5 and 7 post-MI (**b**,**c**). Cellular components markedly decline at day 10 post-MI (**d**). Scale bar: 200 µm (HE staining).

**Figure 2:**
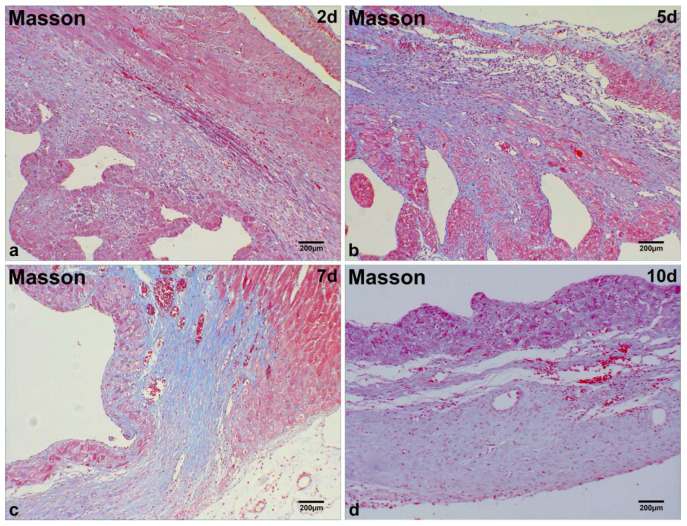
Histological characterization of MI in mice. Degenerated/necrotic myocardial fibers (bluish-purple) dominate the infarct zone at day 2 post-MI (**a**). Fibroblast-like cells infiltrate with interstitial collagen fiber deposition at days 5 and 7 post-MI (**b**,**c**). Increased collagen fiber accumulation in the infarct zoneat day 10 post-MI (**d**). Scale bar: 200 µm (Masson staining).

At day 2 post-MI in mice, significant infiltration of single-nucleated cells and substantial proliferation of fibroblast-like cells were observed in the infarct region (Figure 1a). Degenerated and necrotic myocardial fibers exhibited bluish-purple alterations, as observed through Masson staining (Figure 2a). At days 5 and 7 post-MI, a notable presence of fibroblast-like cells was observed, along with interstitial blue collagen fiber deposits and a limited quantity of regenerating cardiomyocytes in the infarcted region (Figure 1b,c; Figure 2b,c). At day 10 post-MI in mice, an increase in the deposition of blue collagen fibers was observed within the infarct zone, as indicated by Masson staining, alongside a reduction in cellular components (Figure 1d; Figure 2d).

### 2.2. Immunohistochemical Localization of TH, PGP9.5, and SYN in Normal Cardiac Tissue

**Figure 3:**
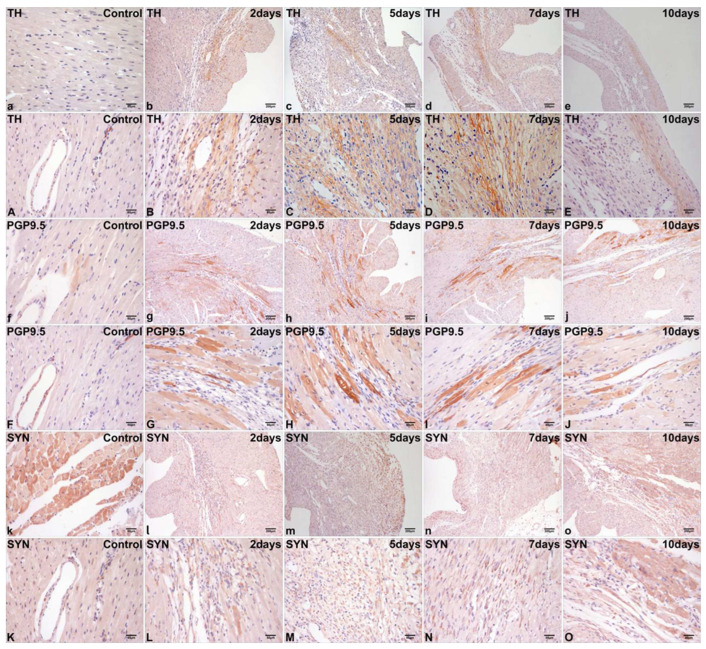
Representative micrographs of immunohistochemical staining for TH (**a**,**A**), PGP9.5 (**f**,**F**), and SYN (**k**,**K**) in normal cardiac tissue. Myocardial upregulation of TH occurred exclusively in sympathetic nerves extending from the peri-infarct to the infarct region at 2 days post-MI (**b**,**B**), 5 days post-MI (**c**,**C**), 7 days post-MI (**d**,**D**), and 10 days post-MI (**e**,**E**) in mice. Positive staining for PGP9.5 was predominantly observed in the regenerating cardiomyocytes within the infarct region and in the peri-infarct cardiomyocytes extending from the peri-infarct to the infarct region at 2 days post-MI (**g**,**G**), 5 days post-MI (**h**,**H**), 7 days post-MI (**i**,**I**), and 10 days post-MI (**j**,**J**) in mice. SYN was primarily localized in the cytoplasm of fibroblast-like cells within the infarct region at 2 days post-MI (**l**,**L**), 5 days post-MI (**m**,**M**), 7 days post-MI (**n**,**N**), and 10 days post-MI (**o**,**O**) in mice. Scale bar: 200 µm/50 µm.

TH was undetected in the normal cardiomyocyte (Figure 3a). PGP9.5 exhibited weakly positive staining localized in the cytoplasm of focal normal cardiomyocytes (Figure 3f). The cytoplasmic expression of SYN in normal cardiomyocytes was significantly higher than that of TH and PGP9.5 (Figure 3k).

TH- and PGP9.5-positive sympathetic nerves exhibited focal distribution in the epicardium, interstitium, and perivascular regions (Figure 3A,F). SYN-positive sympathetic nerves exhibited focal distribution in the interstitium and perivascular regions (Figure 3K).

### 2.3. Differential Immunohistochemical Expression of TH, PGP9.5, and SYN in Post-Infarct Hearts

At 2 days post-MI, immunohistochemical staining mainly demonstrated TH expression in the sympathetic nerves extending from the peri-infarct to the infarct region, characterized by a thin and elongated strip-like morphology (Figure 3b,B). At 5 and 7 days post-MI, a significant presence of TH-positive sympathetic nerves was noted in the infarct region, exhibiting a grid-like morphology (Figure 3c,C,d,D). At 10 days post-MI, TH-positive staining for sympathetic nerves was reduced in the infarct region (Figure 3e,E).

At 2 days post-MI, intense PGP9.5 immunolabeling was primarily localized to cardiomyocytes in both the infarct core and peri-infarct border zone, with sparse signals detected in sympathetic nerves within the infarct region (Figure 3g,G). At 5 days and 7 days post-MI, the quantity of PGP9.5-positive staining for cardiomyocytes within the infarct region and peri-infarct cardiomyocytes exhibited a further increase (Figure 3h,H,i,I). At 10 days post-MI, PGP9.5-positive staining remained present in the cardiomyocytes within the infarct region and in peri-infarct cardiomyocytes; however, the intensity of staining was markedly diminished (Figure 3j,J).

At 2 days post-MI, SYN-positive staining exhibited greater complexity and variability than TH and PGP9.5. SYN was primarily localized in the cytoplasm of fibroblast-like cells within the infarct region (Figure 3l,L). SYN upregulation also occurred in the limited quantities of peri-infarct cardiomyocytes, regenerating cardiomyocytes, and sympathetic nerves within the infarct region. At 5 days and 7 days post-MI, significant immunoreactivity for SYN was predominantly observed in fibroblast-like cells within the infarct region (Figure 3m,M,n,N) and in peri-infarct cardiomyocytes. At 10 days post-MI, there was a tendency for simplified positive immunoreactivity for SYN, primarily observed in peri-infarct cardiomyocytes (Figure 3o,O).

### 2.4. Dynamic Alterations in Myocardial TH, PGP9.5, and SYN mRNA Levels in Post-MI Mice

**Figure 4:**
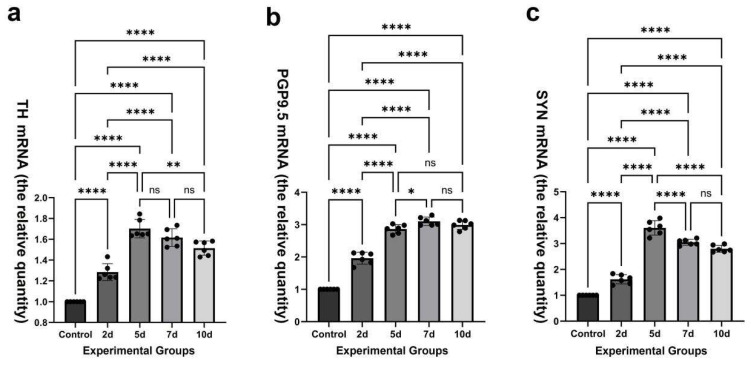
(**a**–**c**) Time-dependent expressions of TH, PGP9.5, and SYN mRNAs in the control group and four experimental groups. The mRNA levels of TH, PGP9.5, and SYN were significantly upregulated from day 2 to day 10 post-MI. Peak expression levels occurred on day 5 for TH (1.702 ± 0.09) and SYN (3.608 ± 0.28) mRNA, and on day 7 for PGP9.5 mRNA (3.102 ± 0.13). All values represent the mean ± SD. **** *p* < 0.0001; ** 0.001 < *p* < 0.01; * 0.01 < *p* < 0.05; ns > 0.05.

In comparison to the control group, the expression levels of mRNA for TH, PGP9.5, and SYN showed significant increases as early as 2 days post-MI and remained elevated until 10 days post-MI (Figure 4). The relative quantities of TH mRNA (1.702 ± 0.09) at 5 days post-MI, PGP9.5 mRNA (3.102 ± 0.13) at 7 days post-MI, and SYN mTNA (3.608 ± 0.28) at 5 days post-MI reached their respective maxima (Figure 4).

Table 1 illustrates that there were no significant differences in the relative quantity of TH mRNA between 5 days post-MI and 7 days post-MI or between 7 days post-MI and 10 days post-MI. No significant differences in the relative quantity of PGP9.5 mRNA were observed between 5 days post-MI and 10 days post-MI or between 7 days post-MI and 10 days post-MI. No significant differences in the relative quantity of SYN mRNA were observed between 7 days post-MI and 10 days post-MI. Significant differences were observed in the relative quantities of TH, PGP9.5, and SYN mRNAs among the other groups.

## 3. Discussion

The current research demonstrates that, in mice, following MI, there is a significant increase in cardiac sympathetic hyperinnervation from the peri-infarct to the infarct region. The temporal progression of sympathetic markers (TH, PGP9.5, and SYN) exhibited a specific increase during the post-infarction phases in the myocardium. TH immunopositivity for sympathetic nerves was observed from the peri-infarct to the infarct region between 2 days post-MI and 10 days post-MI, effectively delineating the morphology of sympathetic nerves with high sensitivity. Furthermore, TH was solely expressed in sympathetic nerves and was absent in other cardiac tissues in both the control group and experimental groups, demonstrating high specificity. PGP9.5 was predominantly observed in the regenerating cardiomyocytes within the infarct region and in peri-infarct cardiomyocytes, while SYN was primarily located in the fibroblast-like cells within the same region. Therefore, this study proposes for the first time that TH functions as a preferred marker for sympathetic localization after MI.

Additionally, the expression of TH, PGP9.5, and SYN mRNAs rose significantly as early as 2 days post-MI, peaking at 5 days post-MI or 7 days post-MI; this was followed by a slight decline observed at 7 to 10 days post-MI, as determined by qRT-PCR analysis. Although the expression trends of TH, PGP9.5, and SYN mRNAs and their corresponding proteins are not entirely synchronous, this discrepancy is likely attributable to the sequential regulation of gene expression: mRNA transcription typically precedes and regulates subsequent protein translation, resulting in a physiological time lag between mRNA and protein peaks. Such staggered expression kinetics are consistent with canonical gene regulatory mechanisms, where transcriptomic changes precede proteomic adaptations in pathological conditions [19].

The expression trends of TH, PGP9.5, and SYN after MI in mice were comparable; however, their impacts on MI were markedly distinct. TH serves as a rate-limiting enzyme in catecholamine biosynthesis, facilitating the conversion of L-tyrosine to L-dihydroxyphenylalanine (L-DOPA) through a tetrahydrobiopterin-dependent monooxygenase reaction. L-DOPA is subsequently converted to dopamine through the action of dopamine decarboxylase and pyridoxal phosphate, which is then further transformed into norepinephrine and epinephrine by dopamine beta-hydroxylase [20]. Norepinephrine binds to β-receptors in cardiomyocytes, accelerating the inward flow of calcium ions during phase 0 of slow-response cells. This increases the rate of the rise in the action potential (AP) in phase 0 and enhances atrioventricular conduction, leading to the occurrence of cardiac MA [21]. Recent research indicates that systemic norepinephrine can elevate ventricular interstitial neuropeptide Y (NPY) levels, implying that norepinephrine stimulates NPY release from postganglionic sympathetic nerves. Elevated NPY levels correlate with heightened ventricular arrhythmias and mortality in heart failure patients [22]. Thus, the up-regulation of TH expression can represent sympathetic nerve hyperinnervation and enhanced sympathetic activity within the infarct region, which likely contributes to the onset of cardiac arrhythmias after MI, as is consistent with our prior study involving human specimens [16]. A recent study in a large animal model has provided compelling functional evidence for this phenomenon [23]. By directly measuring the dynamic release of neurotransmitters within the myocardial interstitium, this research confirmed that the heart exhibits regionally heterogeneous release of both norepinephrine and NPY under stress after myocardial infarction. Furthermore, this abnormal release could be partially suppressed by Axonal Modulation Therapy (AMT) targeting the stellate ganglia. This discovery not only supports the potential of NPY as a crucial biomarker and therapeutic target, but also suggests that combining morphological markers (such as our TH findings) with functional indicators (such as dynamic NPY release) may represent a precise future direction for assessing cardiac sympathetic nerve activity, identifying high-risk patients, and guiding neuromodulation therapies.

While sparse sympathetic nerve fibers retained weak PGP9.5 immunoreactivity, predominant expression was localized to cardiomyocytes of the infarct core and border zone, demonstrating spatial specificity distinct from sympathetic innervation patterns in the study. Macrophages and myofibroblasts exhibited NGF expression in regions adjacent to sympathetic nerves [24]. The local secretion of neurotrophic proteins by these cells may trigger PGP9.5 expression in cardiomyocytes post-MI. PGP9.5 is alternatively known as ubiquitin carboxyl-terminal hydrolase L1 (UCHL1). It is a deubiquitinating enzyme (DUBs; also referred to as deubiquitinase) that removes ubiquitin or polyubiquitin from target proteins [25]. UCHL1 expression was significantly upregulated in cardiomyocytes after MI, correlating with elevated ubiquitin expression [17]. The pathophysiological significance of UCHL1 expression in the cardiomyocytes may stabilize epidermal growth factor receptor (EGFR) through deubiquitination, thereby activating its downstream mediators. The systemic administration of the UCHL1 inhibitor LDN-57444 markedly reversed cardiac hypertrophy and remodeling [26]. This same group later reported analogous findings in the spontaneous hypertensive rat model, utilizing only the pharmacological inhibition of UCHL1 through LDN-57444 [27]. Recent LC3-II flux assays indicate a reduction in autophagic flux in CKO mouse myocardium and in cultured Uchl1-deficient cardiomyocytes. The upregulation of UCHL1 in post-MI hearts may confer protection against cardiac remodeling and dysfunction, potentially by facilitating autophagic flux and maintaining proteostasis under stress conditions [28].

This study revealed that SYN is primarily located in fibroblast-like cells within the infarct region, while the exact mechanism of SYN expression in non-neuronal cells remains unclear. Synaptic vesicles (SVs) in nerve terminals are known to be characterized by their small size. The ectopic expression of SYN, the second-most abundant integral membrane protein of SVs, in fibroblastic cells (COS7 cells) [29], in conjunction with synapsin, a peripheral SV protein that can form macromolecular condensates [30], is sufficient to produce liquid clusters of small exo-endocytic recycling vesicles. These clusters resemble the SVs of synapses in terms of size and molecular composition [31,32]. These studies indicate that SYN, located in fibroblast-like cells within the infarct region, may make a significant contribution to the formation of vesicles within the size range of SVs. In the future, an important question to consider is whether the function of SYN-positive fibroblast-like cells within the infarct region is similar to that of nerve terminals.

## 4. Materials and Methods

### 4.1. Animals and Ethical Procedures

A total of 60 healthy adult C57/BL6 male mice, with weights ranging from 22 g to 28 g, were divided into two groups: a control group consisting of normal mice (*n* = 12) and four experimental groups (*n* = 48). The designed MI ages were distributed at 2 days (*n* = 12), 5 days (*n* = 12), 7 days (*n* = 12), and 10 days (*n* = 12).

All mice were acquired from Beijing Vital River Laboratory Animal Technology Co., Ltd (Beijing, China). The mice were maintained in a 12-h light/dark cycle at 25 °C, with unrestricted access to standard chow and tap water in accordance with institutional guidelines. All animal protocols adhered to the “Principles of Laboratory Animal Care” (National Institutes of Health, Published No. 85-23, Revised 1985), aiming to minimize both the number of animals utilized and their potential suffering. Approval was granted by the Ethical Committee of Institute of Evidence Law and Forensic Science at China University of Political Science and Law.

### 4.2. MI Model

MI was induced through surgical ligation of the left anterior descending coronary artery (LAD) in mice under aseptic conditions, following previously established methods with minor modifications [33]. Briefly, after the induction of anesthesia using ketamine (50 mg/kg) and pentobarbital sodium (50 mg/kg), mice were orally intubated and subsequently connected to a MiniVent Mouse Ventilator (Type 845, Harvard Apparatus) set to 125–150 breaths per minute, adjusted according to body weight (tidal volume 6.4 μL/g, PEEP 5–7 cm H_2_O). Anesthesia was sustained using 1% isoflurane. Heat lamps and heating pads were employed to sustain body temperature at 37 °C. Following hair removal and skin disinfection, a left thoracotomy was conducted in the fourth intercostal space to access the beating heart. The LAD was visualized and ligated with a 6-0 silk suture to induce MI. Successful occlusion was verified through the observation of pallor in the myocardial tissue distal to the ligature, accompanied by left ventricular dyskinesia. The chest was closed using a 4-0 silk suture, and the skin was sealed; subsequently, the trachea cannula was gently removed. Buprenorphine SR (1 mg/kg) was administered immediately post-surgery, prior to placing the mice on a warm surface in a new, clean cage for recovery.

CO_2_ asphyxiation was employed to euthanize the mice on the second day (*n* = 12), fifth day (*n* = 12), seventh day (*n* = 12), and tenth day (*n* = 12) post-operation. Histological examinations and immunohistochemical procedures were conducted using transverse sections of the left ventricles from 6 mice. Myocardial specimens were obtained from the left ventricles of six additional mice, which were equally divided into two blocks for quantitative reverse-transcriptase PCR (qRT-PCR). The myocardial specimen in the control group (*n* = 12) was obtained from the same site.

### 4.3. Histological Examination

Histological slides were prepared with 5 mm sections and stained with hematoxylin–eosin (HE) to analyze granulation tissue parameters with modifications—inflammatory infiltrate, vascular proliferation, fibroblastic proliferation and collagenization. Masson staining was used to evaluate the organization and maturation of collagen fibers.

### 4.4. Immunohistochemical Staining

Briefly, tissue sections were mounted on glass slides coated with APES. The sections underwent deparaffinization in xylene, followed by rehydration through a series of graded alcohols, and they were subsequently heated in 0.01 mol/L sodium citrate buffer (pH 6.0) using a medical microwave oven for antigen retrieval. Hydrogen peroxide (3%) was subsequently applied to quench endogenous peroxidase activity. The sections were incubated with 10% non-immune goat serum to minimize non-specific binding. Tissue sections were incubated overnight at 4 °C with rabbit anti-Tyrosine Hydroxylase monoclonal antibody (dilution 1:200; ab137869, Abcam, Cambridge, UK), rabbit anti-PGP9.5 monoclonal antibody (dilution 1:200; ab108986, Abcam, Cambridge, UK), and rabbit anti-Synaptophysin monoclonal antibody (dilution 1:200; ab32127, Abcam, Cambridge, UK). This was followed by incubation with the Histostain-Plus Kit according to the manufacturer’s instructions (Zymed Laboratories, South San Francisco, CA, USA). The sections were routinely counterstained with hematoxylin. Some sections were incubated with PBS instead of the primary antibody as immunohistochemical controls for immunostaining procedures. Hematoxylin-eosin (HE) staining was performed using conventional methods.

### 4.5. qRT-PCR Analysis

Total RNA was isolated using RNAiso Plus (9108, Takara Biotechnology, Shiga, Japan) according to the manufacturer’s guidelines. The RNA concentration was measured using spectrophotometry, and its integrity was evaluated via agarose gel electrophoresis. Reverse transcription of total RNA (1 µg) was conducted utilizing the PrimeScript^TM^ RT reagent Kit (RR037A, Takara Biotechnology). The primers for the target and reference genes, as outlined in Table 2, were designed and synthesized by Takara Biotechnology. Real-time fluorescence detection was performed with an ABI PRISM^®^ 7500 Real-Time PCR System (Applied Biosystems, Foster City, CA, USA), and quantification was determined using the comparative threshold cycle (Ct) method. The Ct of the duplicate measurements was utilized to determine ΔCt, defined as the difference in Ct values between the target and reference genes. The relative quantity of the product was calculated as fold-induction in experimental groups relative to the control group using the formula 2^−ΔΔCt^, where ΔΔCt = ΔCt (2 days, 5 days, 7 days, or 10 days) − ΔCt (the control group).

### 4.6. Statistical Analysis

Data are presented as means ± standard deviation (SD) and were analyzed using SPSS 17.0. Prior to parametric analysis, all variables (TH, PGP9.5, and SYN) were confirmed to meet the assumptions of normality and homogeneity of variances: Normality was assessed using the Shapiro–Wilk test, with a significance threshold of *p* > 0.05 indicating normal distribution. Homogeneity of variances was verified via Levene’s test, where *p* > 0.05 indicated equal variances across groups.

After confirming these assumptions, a one-way ANOVA was employed to compare TH, PGP9.5, or SYN data between groups. A difference of *p* < 0.05 was deemed statistically significant.

## 5. Conclusions

This study demonstrates that the exclusive sympathetic neuronal expression of TH confers superior specificity for tracking post-MI remodeling compared to pan-neuronal markers. Consequently, TH emerges as a more robust and reliable biomarker for evaluating sympathetic nerve remodeling post-MI than PGP9.5 or SYN. Furthermore, immunohistochemical analysis demonstrated ectopic expression of PGP9.5 and SYN in non-neuronal cells within myocardial infarction tissues. However, due to the inherent limitations of immunohistochemistry as a semiquantitative technique and the absence of functional validation through genetic perturbation models (such as cell-specific knockout), these observations remain speculative and require further mechanistic investigation.

## Figures and Tables

**Table 1:** Relative quantity of TH, PGP9.5, and SYN mRNAs in the control group and four experimental groups.

Groups	Mean (%) ± SD (%) (range %)		
	*TH*	*PGP9.5*	*SYN*
Control	1 ^a^	1 ^a^	1 ^a^
2 days	1.284 ± 0.08 (1.222–1.432) ^a^	1.962 ± 0.19 (1.675–2.126) ^a^	1.618 ± 0.18 (1.390–1.834) ^a^
5 days	1.702 ± 0.09 (1.635–1.843) ^b^	2.864 ± 0.14 (2.675–3.025) ^e^	3.608 ± 0.28 (3.213–3.982) ^a^
7 days	1.616 ± 0.08 (1.523–1.735) ^c^	3.102 ± 0.13 (2.983–3.294) ^e^	3.050 ± 0.12 (2.898–3.212) ^f^
10 days	1.515 ± 0.07 (1.430–1.582) ^d^	2.991 ± 0.13 (2.784–3.132) ^d^	2.799 ± 0.13 (2.674–2.984) ^g^

^a^ *p* < 0.0001 (vs. each group), ^b^ *p* < 0.0001 (vs. the control and 2 days post-MI group) and 0.001 < *p* < 0.0001 (vs. 10 days post-MI group), ^c^ *p* < 0.0001 (vs. the control and 2 days post-MI group), ^d^ *p* < 0.0001 (vs. the control and 2 days post-MI group) and 0.001 < *p* < 0.0001 (vs. 5 days post-MI group), ^e^ *p* < 0.0001 (vs. the control and 2 days post-MI group) or 0.05 (vs. 7 days post-MI group or 5 days post-MI group), ^f^ *p* < 0.0001 (vs. the other groups except the 10 days post-MI group), ^g^ *p* < 0.0001 (vs. the other groups except the 7 days post-MI group).

**Table 2:** qRT–PCR primer sequences.

Gene	Species	Primer		GenBank Accession No.
*Gapdh*	mouse	Forward:	5′-GGTGAAGGTCGGTGTGAACG-3′	NM_017008.3
		Reverse:	5′-CTCGCTCCTGGAAGATGGTG-3′	
*TH*	mouse	Forward:	5′-GTTTCAGTGCACACAGTACATC-3′	NM_009377.2
		Reverse:	5′-CACCGTGGAGAGTTTTTCAATT-3′	
*PGP9.5*	mouse	Forward:	5′-ATAGAGCCAAGTGTTTCGAGAA-3′	NM_011670.2
		Reverse:	5′-ATTCACTTTGTCATCTACCCGA-3′	
*SYN*	mouse	Forward:	5′-CCACTGACCCAGAGAACATTAT-3′	NM_009305.2
		Reverse:	5′-CTTGAACACGAACCATAGGTTG-3′	

*Gapdh*, glyceraldehyde-3-phosphate dehydrogenase; *TH*, tyrosine hydroxylase; *PGP9.5*, protein gene product 9.5. *SYN*, synaptophysin.

## Data Availability

The original contributions presented in this study are included in the article. Further inquiries can be directed to the corresponding author.
